# Biomechanical Assessment of Three Osteosynthesis Constructs by Periprosthetic Humerus Fractures

**DOI:** 10.1155/2020/8872419

**Published:** 2020-10-26

**Authors:** Afif Harb, Bastian Welke, Emmanouil Liodakis, Sam Razaeian, Dafang Zhang, Christian Krettek, Christof Hurschler, Nael Hawi

**Affiliations:** ^1^Trauma Department, Hannover Medical School (MHH), Carl-Neuberg-Str. 1, Hannover 30625, Germany; ^2^Laboratory for Biomechanics and Biomaterials, Department of Orthopaedic Surgery, Hannover Medical School, Hannover, Germany; ^3^Department of Orthopaedic Surgery, Brigham and Women's Hospital, 75 Francis St, Boston, MA 02115, USA

## Abstract

**Background:**

Biomechanical stability assessment of 3 different constructs for proximal fixation of a locking compression plate (LCP) in treating a Worland type C periprosthetic fracture after total shoulder arthroplasty.

**Methods:**

27 Worland type C fractures after shoulder arthroplasty in synthetic humeri were treated with 14-hole LCP that is proximally fixed using the following: (1) 1 × 1.5 mm cerclage wires and 2x unicortical-locking screws, (2) 3 × 1.5 mm cerclage wires, or (3) 2x bicortical-locking attachment plates. Torsional stiffness was assessed by applying an internal rotation moment of 5 Nm and then after unloading the specimen, an external rotation moment of 5 Nm at the same rate was applied. Axial stiffness was assessed by applying a 50 N preload, and then applying a cyclic load of 250 N, then increasing the load by 50 N each time, until a maximum axial load of 2500 N was reached or specimen failure occurred.

**Results:**

With regard to internal as well as external rotational stiffness, group 1 showed a mean stiffness of 0.37 Nm/deg and 0.57 Nm/deg, respectively, group 2 had a mean stiffness of 0.51 Nm/deg and 0.39 Nm/deg, respectively, while group 3 had a mean stiffness of 1.34 Nm/deg and 1.31 Nm/deg, respectively. Concerning axial stiffness, group 1 showed an average stiffness of 451.0 N/mm, group 2 had a mean stiffness of 737.5 N/mm, whereas group 3 had a mean stiffness of 715.8 N/mm.

**Conclusion:**

Group 3 displayed a significantly higher torsional stiffness while a comparable axial stiffness to group 2.

## 1. Introduction

Periprosthetic fractures occur in approximately 0.6% to 3% of all total shoulder arthroplasties (TSA) [1]. Since periprosthetic fractures are heterogeneous in nature, several classification systems have been developed to guide their treatment. In our study, we used the Worland classification [2], which classifies periprosthetic humerus fractures into three types, A, B, and C, based on the fracture location and stability of the prosthetic stem. Given the wide-ranging variations in the nature of periprosthetic humerus fractures, a wide array of treatment recommendations exist, either for conservative or surgical treatment [3]. Within the realm of surgical treatment, stabilization of the proximal segment in itself presents a technical challenge due to the presence of the prosthetic stem with or without a cement mantle. This has led to the emergence of an array of options for proximal plate fixation, including cerclage wires or cables, locking or nonlocking unicortical screws, allograft struts, and more recently, plate designs that allow bicortical fixation by directing offset locking screws tangentially around either side of the prosthesis stem [4]. The objective of our study was to perform a biomechanical assessment of torsional and axial stability of the proximal plate fixation in a Worland type C periprosthetic humerus fracture after total shoulder arthroplasty using three different fixation constructs.

## 2. Materials and Methods

### 2.1. Specimens

**Figure 1:**
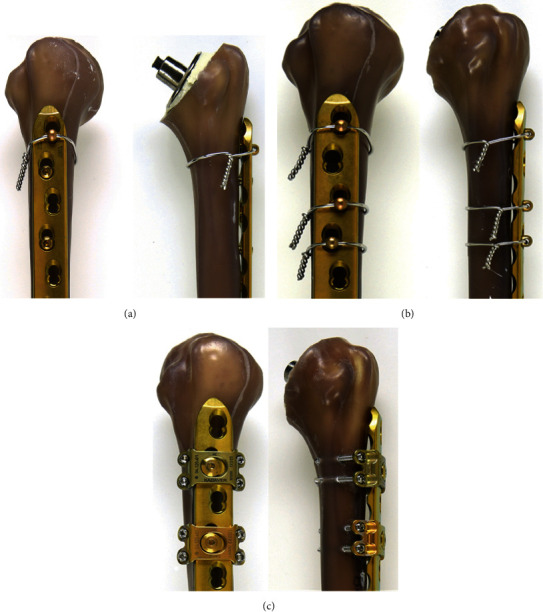
(a). LCP plate fixed on the humerus using 1 × 1.5 mm cerclage wires placed at the level of the 1^st^ hole and 2x unicortical locking screws placed at the levels of the 3^rd^ and 4^th^ holes. (b) LCP plate fixed on the humerus using 3 × 1.5 mm cerclage wires placed at the levels of the 1^st^, 3^rd^, and 4^th^ holes. (c) LCP plate fixed on the humerus using 2x locking attachment plates allowing bicortical fixation placed at the levels of the 2^nd^ and 4^th^ holes.

27 synthetic humeri with left-sided geometry (#3404, 4th generation, Sawbones, Pacific Research Laboratories Inc., Vashon, WA, USA) were used for this biomechanical study. An anatomical total shoulder prosthesis (Univers™ II, Arthrex, Florida, USA) was implanted according to the operating instructions of the manufacturer. Each prosthesis was fixed using 40 g of Refobacin Bone Cement (Zimmer Biomet GmbH, France). After allowing for the cement to harden, a 14-hole 3.5 mm metaphyseal locking compression plate (LCP) (DePuy Synthes, Switzerland) was positioned along the lateral surface of the humeral shaft. The plate was proximally attached to the synthetic bone using three different fixation constructs. Thereafter, the specimens were divided into three different groups with nine specimens in each group:

- LCP attached to the proximal humerus using 1 × 1.5 mm cerclage wires and 2x locking unicortical screws (Figure 1(a))
- LCP attached to the proximal humerus using 3 × 1.5 mm cerclage wires (Figure 1(b))
- LCP attached to the proximal humerus using 2x locking attachment plates allowing bicortical fixation by directing offset locking screws tangentially around either side of the prosthesis stem (Figure 1(c))

**Figure 2:**
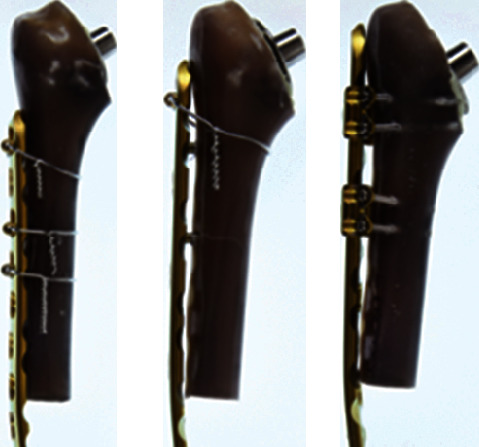
Humerus-LCP construct after performing the transverse osteotomy 5 cm distal to the end of the prosthesis, with the irreversibly bent LCP after performing the biomechanical stability testing.

Following fixation of the LCP onto the proximal segment of the humerus, we performed a 90° transverse osteotomy in the humeral mid-diaphysis 5 cm distal to the end of the prosthesis, simulating a Worland type C periprosthetic fracture and ensuring a stable prosthesis. To focus on the proximal plate fixation and to simulate an unstable fracture situation, only the proximal part of the artificial humerus was used (Figure 2).

### 2.2. Mechanical Testing

**Figure 3:**
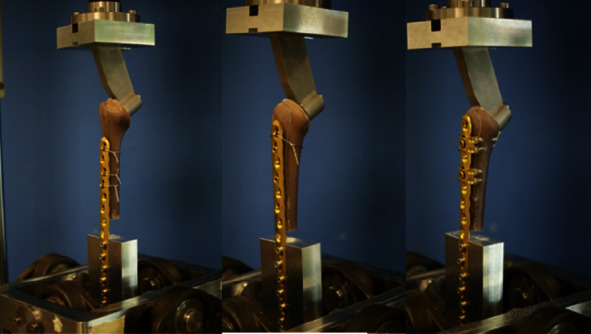
Humerus-LCP construct mounted on the servo-hydraulic material testing machine.

The biomechanical investigations were carried out on a servo-hydraulic material testing machine (MTS MiniBionix I, Model 858, Eden Prairie, Minneapolis) and a custom-made experimental setup. The synthetic humeri were mounted between two universal joints (Figure 3). The distal part of the specimen was directly attached to a mounting block with the LCP. At the proximal end, the specimen was fixed to the upper Cardan joint by means of the taper of the shoulder prosthesis. The loads were applied to the specimen from the proximal end by the actuator of the material testing machine.

### 2.3. Torsional Stiffness Testing

The specimens were axially loaded with a static force (compression) of 5 N with a loading rate of 0.5 N/s. Afterwards, an internal rotation moment of 5 Nm was applied at a rate of 0.5 Nm/s. After reaching the maximum moment, the specimen was unloaded and an external rotation moment of 5 Nm at the same rate was applied. Finally, the machine adjusted the axial torque back to 0 Nm. The specimens were not damaged during the test. The protocol was carried out in load and torque control. Time, cycles, angle, torque, and force were recorded with a sampling rate of 1 kHz.

### 2.4. Axial Stiffness Testing

The axial stiffness investigation was carried out in a cyclic test. Initially, the specimens were loaded with a preload of 50 N. In the first loading stage, a cyclic load with 1 Hz was applied in the sinusoidal form up to a load of 250 N. The lower load in all stages was 0 N, and ten cycles were carried out in each stage. In the following stages, the upper load limit was increased by 50 N each time. Thereafter, the upper load was increased until a maximum axial load of 2,500 N was reached or until the specimen sustained irreversible damage. The protocol was carried out in load and torque control. Time, cycles, angle, torque, and force were recorded with a sampling rate of 1 kHz. Load to failure was assessed by measuring the amount of axial force exerted at the time where the proximal fixation fails.

### 2.5. Load to Failure of the Locking Compression Plate (LCP)

This test was performed to assess the maximum axial load capacity that the LCP could withstand. This test was a pure assessment of the LCP with no additional component attached to it. The plates were fixed in the assembly so that the load being applied onto the plate was identical to the load previously being applied onto both the plate with the humerus fixed to it. In total, three plates were tested with the identical load protocol as for the axial stiffness testing as well as load to failure.

### 2.6. Statistics

Statistical analysis was performed using SPSS software (IBM SPSS Statistics 26.0, SPSS Inc., Chicago, IL). The significance of differences between all three groups was tested using the Kruskal–Wallis test. Significant differences between the two groups were assessed using the Mann–Whitney *U* test. The significance level of *α* = 0.05 was employed.

## 3. Results

### 3.1. Torsional Stiffness

**Figure 4:**
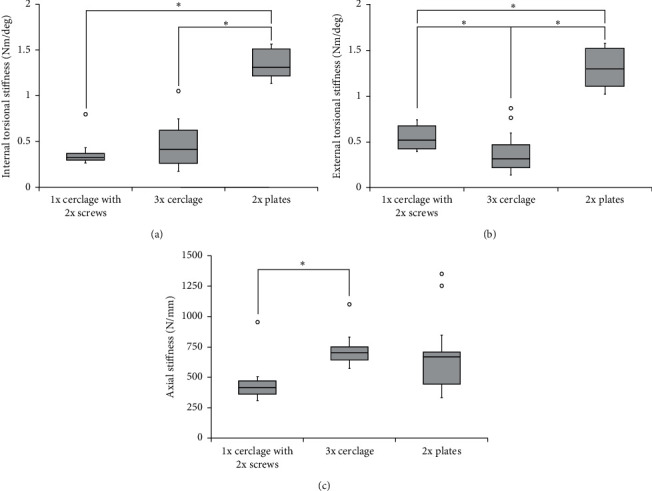
(a) Box plot showing the internal rotational stiffness of the three samples. The fixation with 2x plates provided the highest internal rotational stiffness, followed by both that with 3x cerclage wires and that with 1x cerclage and 2x screws which in turn displayed a somewhat similar stiffness, where the difference between groups 1 and 3 as well as those between 2 and 3 were statistically significant. (b) Box plot showing the external rotational stiffness of the three samples. The fixation with 2x plates provided the highest external rotational stiffness, followed by that with 3x cerclage wires and then by that with 1x cerclage and 2x screws, where the difference between groups 1 and 2, groups 2 and 3, and groups 1 and 3 were statistically significant. (c) Box plot showing the axial stiffness of the three samples. The fixation with 3x cerclage wires provided the highest axial stiffness, followed by that with 2x plates and then by that with 1x cerclage and 2x screws, where the difference between groups 1 and 2 were statistically significant.

After applying a 5 N axial load, the specimens that were treated with 1 × 1.5 mm cerclage wires and 2x locking unicortical screws showed a mean internal torsional stiffness of 0.37 ± 0.15 Nm/deg (mean ± standard deviation), while the specimens treated with 3 × 1.5 mm cerclage wires showed an average internal torsional stiffness of 0.51 ± 0.33 Nm/deg, and those treated with 2x locking attachment plates with bicortical screws showed a mean internal torsional stiffness of 1.34 ± 0.16 Nm/deg (Figure 4(a)). The differences between the groups with the specimens treated with 1 × 1.5 mm cerclage wires and 2x locking unicortical screws and the specimens with the 2x locking attachment plates with bicortical screws were statistically significant (*p* < 0.001). Furthermore, the difference between the specimens treated with 3 × 1.5 mm cerclage wires and those with the 2x locking attachment plates with bicortical screws were statistically significant (*p* < 0.001) (Table 1).

After reaching the maximum moment of each specimen in each group and adjusting the axial torque back to 0 Nm, an external torsional stress was applied allowing for the measurement of external torsional stiffness. The mean external torsional stiffness was 0.57 ± 0.20 Nm/deg for the specimens treated with 1 × 1.5 mm cerclage wires and 2x locking unicortical screws, 0.39 ± 0.24 Nm/deg for the group treated with 3 × 1.5 mm cerclage wires, and 1.31 ± 0.24 Nm/deg for the group treated with 2x locking attachment plates with bicortical screws (Figure 4(b)). The differences between the groups with the specimens treated with 1 × 1.5 mm cerclage wires and 2x locking unicortical screws and the specimens with the 2x locking attachment plates with bicortical screws were statistically significant (*p* < 0.001). Furthermore, the difference between the specimens treated with 3 × 1.5 mm cerclage wires and the specimens with the 2x locking attachment plates with bicortical screws were statistically significant (*p* < 0.001). The difference between the groups treated with 1 × 1.5 mm cerclage wires and 2x locking unicortical screws and 3 × 1.5 mm cerclage wires were statistically significant (*p*=0.043).

### 3.2. Axial Stiffness Testing

Axial stiffness was assessed by implementing a cyclical axial load to reach a maximum of 2,500 N or until the construct failed after applying an initial axial preload of 50 N. The specimens that were treated with 1 × 1.5 mm cerclage wires and 2x locking unicortical screws showed a mean axial stiffness of 451.0 ± 41.6 N/mm, while the specimens treated with 3 × 1.5 mm cerclage wires showed a mean axial stiffness of 737.5 ± 146.7 N/mm, and the specimens treated with 2x locking attachment plates with bicortical screws showed an average axial stiffness of 715.8 ± 357.7 N/mm (Figure 4(c)). The difference between the group treated with 1 × 1.5 mm cerclage wires and 2x locking unicortical screws and the group treated with 3 × 1.5 mm cerclage wires were statistically significant (*p*=0.001).

### 3.3. Patterns of Proximal Fixation or Experimental Setup Failure

When assessing the three LCPs without any humerus attached, the mechanical axial load limit of the LCPs was 1,190 N. From this axial load on, the LCPs were irreversibly deformed. When the specimens with the humeri reached this load limit, the LCPs themselves and not the proximal fixation constructs failed. However, the tests were not completed at this point. The loads were increased further until failure of the proximal shoulder implants or the proximal LCP fixation occurred. In the group where the plates were proximally fixed with 1 × 1.5 mm cerclage wires and 2x locking unicortical screws, the LCPs were irreversibly bent in two specimens, the unicortical screws loosened in five specimens, and the attachment between the shoulder prostheses and the servo-hydraulic material testing machine was disrupted in two specimens. In the group with the 3 × 1.5 mm cerclage wires, cerclage wire loosening or rupture occurred in five specimens, the LCPs were irreversibly bent in three specimens, and the distal part of the humerus came into contact with the base of the servo-hydraulic material testing machine in one specimen. In the group with the 2x locking attachment plates with bicortical screws, the LCP was irreversibly bent in eight specimens, and the distal part of the humerus came into contact with the base of the servo-hydraulic material testing machine in one specimen (Table 2).

## 4. Discussion

Given the heterogeneous nature of these fractures, various surgical techniques have been suggested and used to treat these injuries, including cerclage wiring, plating, and interfragmentary screw insertion. There seems to be a general inclination and recommendation leaning towards surgically treating periprosthetic fractures distal to the prosthetic stem when the prosthesis is stable in order to optimize rates of healing, return to function, and painless mobilization. This strategy has been supported by the works of Bonutti and Hawkins and Boyd et al. in their case series of four and seven patients, respectively, with periprosthetic humeral shaft fractures after TSA [5, 6]. However, despite the general agreement that surgery is the treatment of choice for these injuries, there is no consensus on the best surgical technique. To the knowledge of the authors, there has been no systematic testing of the strength of biomechanical constructs in simulated models of periprosthetic fractures distal to a stable TSA prosthesis to show that one construct is superior to another. In the present study, we have performed a biomechanical assessment of three commonly used surgical constructs for treating Worland type C periprosthetic fractures after TSA.

Multiple biomechanical studies of periprosthetic proximal femur fracture have mechanically loaded both the proximal and distal bone attachment segments together, with many failures occurring during these tests at the level of the distal fixation [7–9]. Distal failures, in turn, make it difficult to draw conclusions about construct stability at the level of the proximal fixation. To address this issue, in the present study, the attachment of the plate to the proximal fragment was tested in isolation, an approach previously described by Lenz et al. [10].

When comparing torsional stiffness, both internal and external, the group treated with the 2x locking attachment plates with bicortical screws displayed significantly higher stability and stiffness compared with the other two groups (*p* < 0.05). This supports the results of Gregory et al. where bicortical screws showed significantly higher torsional strength compared to unicortical screws or cerclage wires alone in the treatment of periprosthetic femur fractures [4]. The groups treated with cerclage wires and unicortical screws and the group treated with cerclage wires alone showed a comparable torsional stability for both internal and external torsional stresses (*p* < 0.05). This result contrasts with the findings of Dennis et al. and Fulkerson et al., which demonstrated that periprosthetic femur fractures treated with unicortical screws displayed a significantly higher torsional stability compared to fractures treated with cerclage wires alone [11, 12].

When comparing axial stiffness, the groups treated with 2x locking attachment plates and bicortical screws and the group treated with cerclage wires alone displayed comparable axial stiffness, both of which were higher than the group treated with cerclage wires and unicortical screws; however, only the difference between the group with both cerclage wires and screws and the group treated with cerclage wires alone was found to be statistically significant (*p*=0.001). Our findings contrast with the findings of Lenz et al. showed that plates with bicortical screws displayed higher axial stability when compared to cerclage wires in periprosthetic fractures of the femur [10]. We believe that this phenomenon could be simply explained by the fact that an initial 50 N axial load was applied on all specimens before initiating the cyclical axial load testing. After this initial load, it was visible to the experimenters that the constructs treated with the cerclage wires in isolation showed a small displacement in which the humeri were moved caudally several mm until the cerclage wires were fixed at a more cranial location on the humeri, where the circumference was in fact larger than the initial circumference at the beginning of the testing process. This may have provided the constructs treated with cerclage wires improved stability at the level of the LCP-humerus interface. Interestingly, in our study, the fractures were treated with cerclage wires alone showed a higher axial stiffness when compared to the fractures treated with both cerclage wires and unicortical screws. This finding contrasts with the findings of Lenz et al., where periprosthetic femur fractures treated with 1 cerclage cable and multiple unicortical screws displayed higher axial stability compared with fractures treated with cerclage cables alone [13].

It is worth mentioning that, in our study, the group treated with 2x locking attachment plates and bicortical screws displayed a wider SD range when assessing axial stiffness compared to that of the other two groups. This phenomenon may be due in part to differences in the distance between the humeral transverse osteotomy and the proximal edge of the servo-hydraulic press across specimens, which in turn may be resulted in variable lever arms and a wider standard deviation than expected.

The present study has several limitations in addition to the small sample size. Experiments were conducted on synthetic humeri, whose properties differ from those of *in vivo* samples. Some complexities in humeral loading were not considered. Loads not acting through the shoulder joint, from muscles and other soft tissues, were, for example, not assessed. The distal segment of the humerus was not considered in the model, precluding failures of this segment in the testing. Moreover, we did not assess the amount of displacement that each fixation method may have displayed before the failure of the setup. Nonetheless, this study provided novel information that was scarcely assessed previously; while biomechanical testing has been previously performed in models of periprosthetic fractures of the femur after total hip arthroplasty, research on periprosthetic humerus fractures after TSA is almost nonexistent. This might be due to the fact that hip arthroplasties have been present since 1925 when the American surgeon Marius Smith-Petersen created the first mold arthroplasty out of glass [14]. Even though the first-ever reported TSA took place in France in 1893 by the French surgeon Jules Emile Péan [15], it is only recently that both surgeons and patients have accepted TSA as a good therapeutic option. Accordingly, TSA usage is increasingly growing, allowing the implants and implantation techniques to evolve, and the number of TSA as well as periprosthetic fractures after TSA to increase. Accordingly, it is the opinion of the authors that more biomechanical studies are needed to guide optimal construct choice and surgical treatment for periprosthetic fractures after TSA.

## 5. Conclusion

Locking attachment plates with bicortical locking screws provide significantly better torsional stability compared with cerclage wires alone or cerclage wires in combination with locking unicortical screws in the treatment of Worland type C periprosthetic fractures of the humerus after TSA. However, cerclage wires alone provide an economically cheaper alternative while providing comparable axial stability to its more expensive locking plate counterpart. Nonetheless, further biomechanical studies on periprosthetic humerus fractures after TSA are needed to help guide treatment of this increasingly common and challenging injury.

## Figures and Tables

**Table 1:** An overview table of the results in terms of mean value and standard deviation for each of the three groups.

	Cerclage + unicortical locking screws	Cerlcage	Plate + bicortical locking screws
Internal torsional stiffness (Nm/°)	0.37 ± 0.15	0.51 ± 0.33	1.34 ± 0.16
Mean ± SD			

External torsional stiffness (Nm/°)	0.57 ± 0.20	0.39 ± 0.24	1.31 ± 0.24
Mean ± SD			

Axial stiffness (N/mm)	451.0 ± 41.6	737.5 ± 146.7	715.8 ± 357.7
Mean ± SD			

**Table 2:** An overview of the mechanisms of failure in each of the three groups.

	Cerclage + unicortical locking screws	Cerclage	Plate + bicortical locking screws
Loosening or rupture of the cerclage wire		5	
Irreversibly bent plate	2	3	8
Loosening or screw pullout	5		
Loosening or damage of the prosthesis	2		
Distal humerus end in contact with the hydraulic machine		1	1

## Data Availability

The statistical data used to support the findings of this study are available from the corresponding author upon request.
